# Supplementary figures and images for: A large and unusually colored new snake species of the genus Tantilla (Squamata; Colubridae) from the Peruvian Andes
Source: PeerJ. 2016 Dec 13;4:e2767. doi: 10.7717/peerj.2767 (PMC5157193; doi:10.7717/peerj.2767)

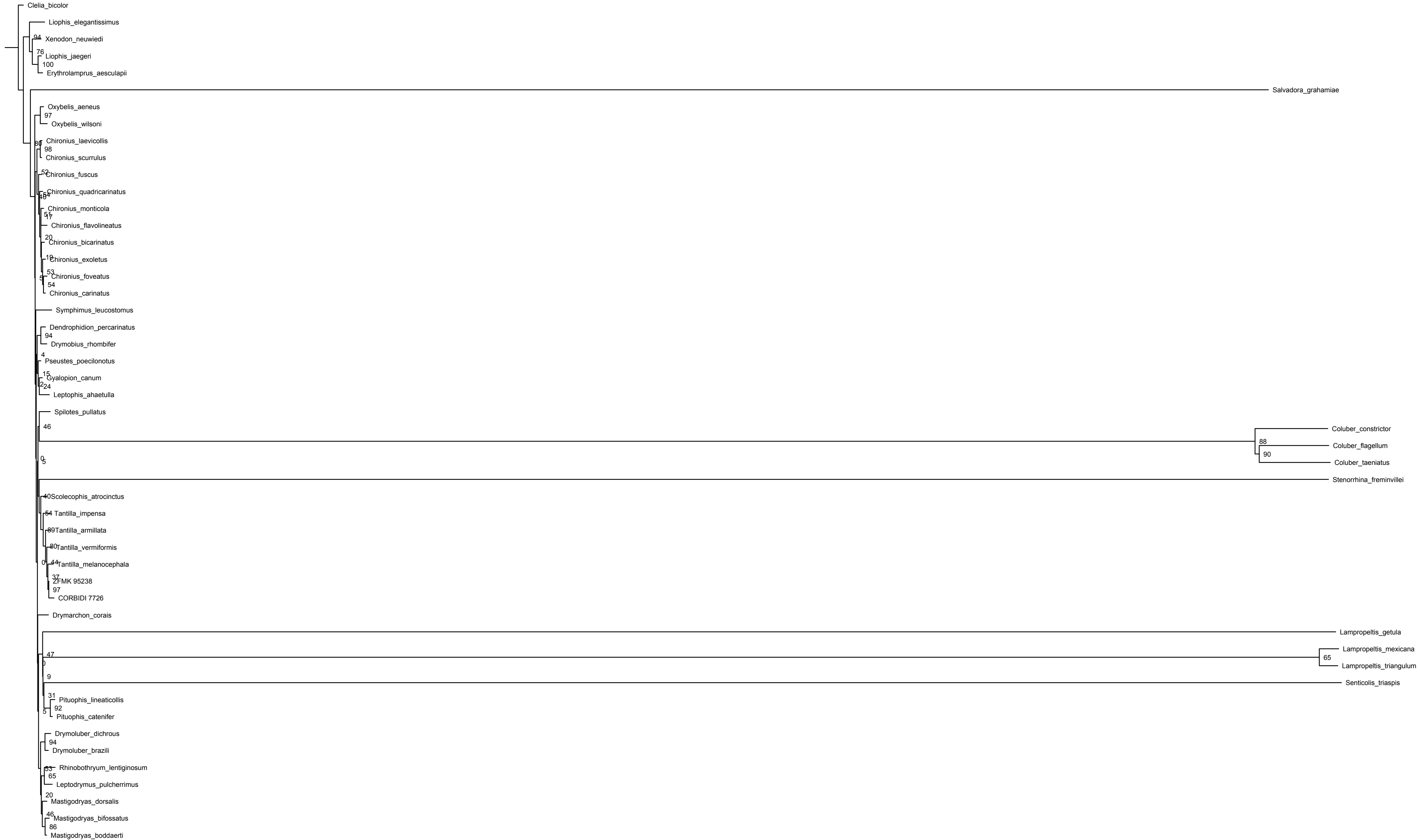

Supplement: Figure S1 [file peerj-04-2767-s007.pdf]
